# Materia Medica

**Published:** 1858-11

**Authors:** 


### MATERIA MEDICA.

XIV.—*Argenti Nitras in Oxyuris Vermicularis.* By Dr. C. H. Schultz.

The author ordered enemata of Argent. Nitr. crystal. grs. x–xv, to aq. destil. ℥iv, and cured his patients, with two or three injections of this kind, completely, and without trouble. The first injection does usually not remain long, and with it many partly dead, partly living worms, are discharged. The subsequent clysters, however, remained six to twenty-four hours, and a great number of dead worms were evacuated with them.—(*Deutsche Klinik.*, 17. 1858.)

XV.—*Iodide of Potassium as Antigalactic.* By Prof. Rousset.

The troublesome milk-knots which like to appear especially at the commencement of lactation, giving rise to fever, inflammation of the breast, and abscesses, indicate a diminution of the secretion of milk by therapeutic means. As the usual measures, emollient cataplasms, dieting, and laxatives, are frequently insufficient, the author tried the iodide of potassium with the following results: The iodide of potassium occasions a considerable decrease of the milk, and in consequence of it prevents and removes milk-knots, particularly if at the same time the child is not put to breast. The milk returns quickly, if the medicine is not used any longer than two to three days; its effect is more decided if the dose does not exceed forty to fifty centigrmm. daily. The secretion of milk can be prevented almost completely if the iodide of potassium is given on the first or second day after delivery. The author gives a full report of seven cases to confirm the above statements.—(*Journal de Bordeaux,* May, 1858.)

XVI.—*Cerate of Opium in Carbuncle.* By DR. W. VON GUTZEIT.

A cerate containing one-half drachm of opium to two ounces of simple cerate, is spread thickly upon linen and applied to the swelling and its neighborhood. This application diminishes pain quickly, generally in about half an hour, hastens suppuration, the detachment of the slough, and the cicatrization of the suppurating surfaces, and ameliorates the general condition of the patient. No medicine was given internally. The opiate cerate can be used at any stage of the disease, and its curative effect seems to surpass that of every other known remedy.—(*Medic. Ztg. Russl.*, 12, 1848; *Schmidt's Jahrbücher*, 8, 1858.)

